# Pisciculture—External and Artificial Fecundation of Fish—Repeopling of the Waters of France

**Published:** 1853-09

**Authors:** 


### *Pisciculture—External and Artificial Fecundation of Fish—Repeopling of the Waters of France.*

M. Coste, in commencing his course on Embryology yesterday, at the College of France, gave an interesting and graphic account of the mode of artificial production of fish. He has also made a detailed report on the subject to the French Institute. It is well known that this has become, within the last few years, quite an important branch of human industry, and that it will afford an efficient means of supplying mankind with food. To the physiologist and man of science, it offers, of course, a means for the study of embryonic development in all its stages, which is of great value. Government here very wisely assists by its capital at the inception of all attempts of this kind, for few private individuals can afford to risk their means on experiments. It employs, probably, at some pecuniary loss in the beginning, the most competent persons to superintend. By this enlightened and judicious course of policy, the most rapid progress is made toward the extension of human knowledge and enterprise. The French Government has, in obedience to this, actually created and placed in successful operation, extensive establishments for supplying fish in enormous quantities, and when sufficient time shall be allowed, the results will be astounding. Fisheries have, heretofore, been destructive processes; the present mode of obtaining these animals is entirely productive.

M. Coste has recently been commissioned to examine and report upon the Institution at *or near Huningue*, under the direction of M. Berthot and Detzem, to whom a credit of 30,000 francs are extended. He commenced by remarking that though persons had frequently observed how independent was the generative process existing between the male and female of fish, yet that the Count de M. was the first, a century since, actually to reproduce them by the artificial mixture of the sperm of the one and the egg of the other. The female prepares her bed, deposits her eggs, and the male passing over the same spot, ejects upon them his sperm. M. by constructing a peculiar shaped box, which would protect other animals from preying upon the eggs inclosed, and also serve at the same time to prevent the deposit of too much sediment, was enabled to place this in a running stream where the the male could impregnate the inclosed ova. He thus obtained, after a sufficient lapse of time young fish completely formed, and he published an account of his researches and experiments, but which remained locked up in the cabinets of the learned. One or two others, however, did repeat them, but it has been at a very recent period that a practical application on an extensive scale has been made of it.

The fish which are selected to be reproduced, are the trout and salmon. The female of the former is held by the head with the left hand, and with the right embracing it like a ring, a gentle squeezy motion is made from the head to the anus. Large species, such as salmon, require an assistant, who suspends them by the head, or with a thong passed through the gills, whilst the other makes the usual pressure, but with both hands from the mouth to the verge. By this means the ova are forced out into flat vessels of wood or earth, in which is placed very pure and limpid water. It is necessary that the egg should be perfectly matured, or they prove abortive. To insure and test this is very simple. The fish are taken in the months of October or November, and if when submitted to the pressure the germinative matter is easily forced out, and appears of a glary consistence, it is found to be fit for impregnation. The male is treated pretty much in the same way, and the water which contains the vesicles of the one and the seminal fluid of the other is stirred, so that as complete imbibition as possible can take place. Without this care only the eggs on the upper surface would be exposed to the action of the male fluid. It is also found advisable, as it were to wash or change the water, so as to render it as pure as possible previous to adding the product of the male to that of the female. The vessel containing them is placed in a stream of water and its contents protected from the encroachments of other fish, including even their own species, as well as from being covered with deposit, sand, &c. The male will protect his own progeny, but feeds on that of others of his own species. One male can be used for several successive days, thus allowing some economy in the number required to be kept. One of the locations selected for the sight of the establishment—for there are large buildings attached to it, engravings of which were shown to us—is one of the rivers in the south of France. There is a conduit passing from this, in which the vessels containing the spawn are deposited in order to complete their development and growth. This conduit is divided off by parallel septa, which allow the supervisors to inspect, from time to time, the ova, and preserve them from deposit. By means also of trunks, the amount of water allowed to pass is increased or lessened at pleasure. After attaining a certain size, the water containing them is passed into a kind of expansion or basin of the outlet. This has in the sides a set of arched compartments or boxes, with false bottoms, which the young fish seek by instinct. When it is desired, these are brought together and form a large floating boat. By this arrangement they can be wafted easily along the course of the stream, then separated at pleasure, and their contents deposited in any of the waters of France communicating with that in which the process commenced. Strange to say, it was from Roman remains that the idea was obtained of separating the reservoir into cavities. They were aware of the proclivity of fish to seek the most concealed portions of the basins which held them; and in their rearing of the fish, to which they devoted much attention, they constructed arches very similar to those it is now found expedient to have. Their remains are found near Mount Pausilippo at the present day. Lucullus, Pollion, and many others of the wealthy epicures, filled their ponds with choice foreign fish, and the best modes of rearing them were pursued which the knowledge of that age afforded.

It is intended to introduce the salmon and sturgeon in those waters of France where they are not now found, as the former, for instance, in the Rhone. Fish generally return the succeeding spring to the place where they are spawned. M. Coste remarked that by the immense quantities of these fish which the artificial means of reproduction will furnish, they will even be able to add materially to the fish in the seas where the streams empty. The mullet, and many other approved varieties, will be experimented with, and the markets of the country supplied with them at a very reduced price.

After the lecture, which was illustrated by diagrams, we were invited into an adjoining room of the College of France, and we there actually saw the entire process in miniature. Several troughs, a few inches deep and six or eight broad, by fifteen inches in length, were filled with water kept pure by bits of charcoal; from thence it percolated slowly into others of the same shape and dimensions, in which were placed the eggs. These were kept near the surface by a layer of stems of straw. In a third, were eggs in a more advanced state of development; and, in a fourth, were seen the young fish swimming freely about.

The above series were placed each below the other, so that the water which flowed from the first box supplied in a continued stream those lower, either overrunning the one to reach the others, or passing slowly through their bottoms. Nothing could be more interesting. There were upward of ten thousand, just hatched, contained in the small apparatus we have described. The different stages of progressive development which are thus placed under the eye of the physiologist, will no doubt be exceedingly fruitful in results, and an embryologist like M. Coste, is well selected by government to supervise such an undertaking. In two subsequent lectures, an account was given of the rearing of fish in the celebrated Lagoons in Tuscany, to which they resort in immense quantities to deposit their eggs, and where they are retained by artificial means. These M. C. had visited, and he alluded to the good health of the inhabitants, who, for more than a century, have lived almost exclusively on these animals. F. P. P.

—*Charleston Medical Journal and Review.*

